# Electronic cigarette knowledge, attitudes and use among students at a university in Hangzhou, China

**DOI:** 10.18332/tid/144230

**Published:** 2022-01-28

**Authors:** Juan Fang, Jianping Ren, Lixian Ren, Wendy Max, Tingting Yao, Falin Zhao

**Affiliations:** 1 School of Public Health, Hangzhou Normal University, Hangzhou, China; 2 Institute for Health and Aging, School of Nursing, University of California San Francisco, San Francisco, United States

**Keywords:** university students, knowledge, attitude, electronic cigarette, China

## Abstract

**INTRODUCTION:**

Electronic cigarettes are increasingly popular worldwide, especially among youth. There is growing evidence of the negative health consequences of vaping. Our objective was to assess university students’ knowledge and attitudes regarding electronic cigarettes (e-cigarettes), their use, as well as the associated influencing factors for their use.

**METHODS:**

The study involved an online cross-sectional survey conducted between November 2019 and March 2020 in a university in Hangzhou, China. A total of 563 students completed the questionnaire. Descriptive statistics were used to assess characteristics, knowledge, and attitudes; t-tests, χ^2^-tests and logistic regression models were used to identify factors associated with ever e-cigarette use.

**RESULTS:**

In all, 59.9% of respondents were female and the average age was 20.38 years (SD=1.32). Only 42.6% of the respondents thought that e-cigarettes contain nicotine, 31.1% thought e-cigarettes are tobacco products, and 8.2% of the students reported being ever e-cigarettes users. In regard to attitude, the average score of the students in the Safety dimension was 3.34 (SD=0.64), followed by the Restriction dimension (Mean=2.66, SD=0.83). Correlates of ever use included regions, friends’ and roommates’ ever e-cigarette use, and higher attitude score in the Supervision dimension.

**CONCLUSIONS:**

The university students’ level of knowledge regarding e-cigarettes was not high, and their attitudes regarding e-cigarettes were not that supportive. Students’ ever use of e-cigarettes at a university in Hangzhou was higher than for university students in other cities in China, but lower than for those in foreign countries.

## INTRODUCTION

Electronic cigarettes (e-cigarettes), also called vapes, are popular nicotine delivery devices. The systems are usually powered by a battery, heating a liquid solution that mostly contains nicotine into an inhalable aerosol or vapor^1^. E-cigarettes were originally designed and marketed as a smoking cessation aid. However, e-cigarettes have some potential harms on the health of users. First, nicotine is highly addictive and may have a detrimental impact on brain development in youth^2^. Second, vapor from e-cigarettes has also been shown to contain toxicants^3^ that may lead to cancer^4^. Additionally, exploding batteries can also be harmful^5^.

Although the role of e-cigarettes in smoking cessation is still a controversial public health issue, e-cigarettes are increasingly popular worldwide, especially among youth^6-9^. In the United States, rates of e-cigarette use surpassed those of cigarettes among youth in 2014^2^, and e-cigarette use is a national epidemic with high prevalence among high school and middle school students (27.5% and 10.5%, respectively)^10^. According to a national survey in New Zealand, 40.5% of university students indicated that they had ever used e-cigarettes^7^. A study showed that vaping was 1.5 times higher among university students compared to the general population in Austria, with 32.4% of the students reporting having tried vapes at least once^11^. At a Saudi university, 43.2% of dental students had ever used an e-cigarette^8^. In China, 4.6% of university students in Shanghai who were surveyed in 2018 had used e-cigarettes at least once^12^. In addition, the prevalence rates of e-cigarette use among students who were at college or university in Shanghai in 2017 and Shandong in 2015 were 7.7% and 4.0%, respectively^13,14^.

Regarding knowledge about and attitudes regarding the use of e-cigarettes in university students, the situation is not ideal. Previous research has indicated that 21.6% of the university students in Shanghai, China, thought e-cigarettes have carcinogens, and 63.1% thought that e-cigarettes were less addictive than conventional cigarettes^12^.

With the rapid increase of e-cigarette use among university and college students, there is an urgent need to understand the factors associated with use. It was reported that university students who were males and cigarette smokers were more likely to use e-cigarettes^7,12^. Peers’ e-cigarette use was another factor^12^. It was also suggested that incorrect knowledge and attitudes may lead to wider use of e-cigarettes among college and university students^8,15^.

Since 2018, China has implemented strict national regulations and policies to prevent juveniles from using e-cigarettes. Further, China banned online sales of e-cigarettes on 1 November 2019^16^. In 2021, a clause that e-cigarettes and other new tobacco products shall be implemented with reference to the relevant provisions on cigarettes was added in the Regulations on the Implementation of the Law on Tobacco Monopoly of the People’s Republic of China. However, there is limited information on the characteristics of Chinese e-cigarette users and trends in e-cigarette use among university students, especially among students at Normal University, a teacher-training university. Therefore, more studies that comprehensively examine university students’ specific knowledge about and attitudes regarding e-cigarettes, and more data on e-cigarette use among university students in China are needed.

Our study aimed to estimate the prevalence of ever and current e-cigarette use among university students and to evaluate potential correlates of e-cigarette use including student sociodemographic characteristics, knowledge about and attitudes toward e-cigarettes.

## METHODS

### Research design and participants

We conducted a cross-sectional analysis that utilized a self-report anonymous questionnaire completed between November 2019 and March 2020. The survey aimed to recruit a minimum of 527 participants. This estimate was based on a sample of 4.6% of the university students in Shanghai that had used e-cigarettes at least once in their lifetime, and margin of error of 3%. We also considered a survey rejection rate of 20%. A stratified random sample of 589 registered undergraduates at Hangzhou Normal University received an online survey through a platform (https://www.wjx.cn/) with their consent. There were 75 majors in the university. Every major was coded and five majors (Science, Engineering, Medicine, Management Science and Arts) were selected randomly through the Statistical Package for the Social Sciences (SPSS) version 22.0. All the classes were then coded according the year in the university among the selected majors. A class as the unit was randomly selected for each grade and each major by the same software. Finally, 563 completely filled in questionnaires were available with a response rate of 95.6%.

### Survey measures

The questionnaire was divided into five parts: basic information, e-cigarette use of family and friends, knowledge of e-cigarettes, attitudes about e-cigarettes, and e-cigarette use.

Basic information included demographic characteristics and ever conventional cigarette use. Demographic characteristics included gender, age, ethnic group (Han, ethnic minority), year in school (first, second, third and fourth), major, urban/rural residence, parents’ educational level (primary or less, secondary, tertiary), and per capita household monthly income: 0–5000, 5001–10000, >10000 RMB (1000 Chinese Renminbi about US$160).

Ever conventional cigarette use was ascertained by: ‘Have you ever smoked cigarettes at least once in your lifetime?’, with response options of ‘Yes’ and ‘No’.

The second part of the questionnaire included ever use of e-cigarettes by father, mother, siblings, friends, and roommates, which were ascertained by dichotomous (yes/no) questions: ‘Has your father ever smoked cigarettes at least once in his lifetime?’, ‘Has your mother ever smoked cigarettes at least once in her lifetime?’, ‘Have your siblings ever smoked cigarettes at least once in their lifetime?’, ‘Have your friends ever smoked cigarettes at least once in their lifetime?’, and ‘Have your roommates ever smoked cigarettes at least once in their lifetime?’.

Knowledge of e-cigarettes was assessed with four questions (yes /no/unclear): ‘Do e-cigarettes contain nicotine?’, ‘Are e-cigarettes tobacco products?’, ‘Are e-cigarettes carcinogenic?’, and ‘Are e-cigarettes addictive?’.

We developed 19 items rated on a five-point Likert scale (1=strongly disagree to 5=strongly agree) to evaluate the students’ attitudes about e-cigarettes based on literature review and expert consultation. We used principal component analysis to group these items into five domains: Accessibility (2 items about how accessible e-cigarettes are to students, mainly regarding price and store), Acceptability (2 items about whether the use of e-cigarettes is socially accepted), Safety (4 items about how safe e-cigarettes are regarding health), Supervision (5 items about whether minors should have restricted access to e-cigarettes), and Restriction (6 items about whether the advertisement online for e-cigarettes and sale online of e-cigarettes should be prohibited). Confirmatory factor analysis confirmed good model fit for the scale. (Supplementary file). A total score for each domain was calculated from the average of all items for individuals. The higher the score, the more positive and supportive the attitude toward e-cigarettes. The Cronbach’s alpha for the scale was 0.815 and the test-retest reliability of the scale was highly significant (intraclass correlation coefficient was 0.85). The score of the total scale and five domains were used to explore the associations between students’ attitudes about e-cigarettes and e-cigarette use.

The last part of the questionnaire included four dichotomous (yes/no) questions to assess the prevalence of e-cigarette use among university students. Participants were asked: ‘Have you ever used an e-cigarette in your lifetime?’, ‘Have you used an e-cigarette in the past year?’, ‘Have you used an e-cigarette between 1 and 29 of the past 30 days?’, ‘Have you used an e-cigarette on all of the past 30 days?’. If they answered ‘yes’ to the first, third and fourth question, they were classified as ‘ever users’, ‘current non-daily users’ and ‘current daily users’, respectively.

We conducted a pilot test with 40 students – 20 males and 20 females – at the same university by convenience sampling in October 2019, including 10 freshmen, 10 sophomores, 10 junior and 10 senior students. We made adjustments to the unclear items that students identified during the pilot test.

### Statistical analysis

SPSS (version 22.0) was used to perform analyses. Descriptive statistics including mean and standard deviation (SD) for quantitative variables, and frequency and percentage (%) for qualitative data. Individuals who had ever used an e-cigarette were regarded as ever users, and those who had never used e-cigarettes as non-users. Independent sample t-tests were performed to compare attitudes about e-cigarette use between ever users and non-users. We used χ^2^ tests to compare the prevalence of e-cigarette use by demographics and other characteristics. As e-cigarette use was a dichotomous measure, a binary logistic regression model was selected to explore the motivational factors. In logistic regression analyses, ever e-cigarette use was the dependent variable. Covariates in the logistic model included the significant variables except for the variables with sparse data, which may lead to large odds ratio and wide confidence interval. Two-sided p<0.05 and p<0.01 were considered statistically significant.

## RESULTS

### Participant characteristics

Of the participants, 59.9% were female and most were ethnic Han (95.7%). The average age of the 563 university students was 20.38 years (SD=1.32). More than half of the fathers and mothers of the students had received secondary education, 55.3% and 58.4, respectively. There were 11.2% of the respondents reporting ever conventional cigarette use. Among fathers, mothers, siblings, friends, and roommates, those who had the highest rate of ever vaping were roommates, accounting for 14.9%. Table 1 shows participant demographics and other characteristics.

**Table 1:** Characteristics of university students, Hangzhou, China 2019–2020 (N=563)

*Characteristics*	*n (%)*
**Gender**	
Male	226 (40.1)
Female	337 (59.9)
**Ethnic group**	
Han	539 (95.7)
Ethnic minority	24 (4.3)
**Year in school**	
First	174 (30.9)
Second	142 (25.2)
Third	153 (27.2)
Fourth	94 (16.7)
**Major**	
Science	120 (21.3)
Engineering	91 (16.2)
Medicine	117 (20.8)
Management Science	77 (13.7)
Arts	158 (28.1)
**Father’s educational level**	
Primary or less	61 (10.8)
Secondary	311 (55.3)
Tertiary	191 (33.9)
**Mother’s educational level**	
Primary or less	85 (15.1)
Secondary	329 (58.4)
Tertiary	149 (26.5)
**Regions**	
Urban	304 (54.0)
Rural	259 (46.0)
**Per capita household monthly income** (RMB)	
0–5000	187 (33.2)
5001–10000	239 (42.5)
>10000	137 (24.3)
**Conventional cigarette use**	
Yes	63 (11.2)
No	500 (88.8)
**Mothers’ ever e-cigarette use**	
Yes	6 (1.1)
No	557 (98.9)
**Fathers’ ever e-cigarette use**	
Yes	44 (7.8)
No	519 (92.2)
**Siblings’ ever e-cigarette use**	
Yes	29 (5.2)
No	534 (94.8)
**Friends’ ever e-cigarette use**	
Yes	65 (11.5)
No	498 (88.5)
**Roommates’ ever e-cigarette use**	
Yes	84 (14.9)
No	479 (85.1)

RMB: 1000 Chinese Renminbi about US$160.

### Knowledge about e-cigarettes

As shown in Table 2, approximately two-thirds of students were aware of the addictiveness of e-cigarettes, and 51.0% participants considered that e-cigarettes are carcinogenic. However, fewer than half of participants (42.6%) were sure that e-cigarettes contain nicotine. In addition, fewer than one-third of students (31.1%) identified e-cigarettes as tobacco products.

**Table 2:** Knowledge regarding e-cigarettes among university students, Hangzhou, China 2019–2020 (N=563)

*Measures*	*Yes n (%)*	*No n (%)*	*Unclear n (%)*
Do e-cigarettes contain nicotine?	240 (42.6)	113 (20.1)	210 (37.3)
Are e-cigarettes tobacco products?	175 (31.1)	225 (40.0)	163 (29.0)
Are e-cigarettes carcinogenic?	287 (51.0)	79 (14.0)	197 (35.0)
Are e-cigarettes addictive?	372 (66.1)	47 (8.3)	144 (25.6)

### Attitudes towards e-cigarettes

Self-reported attitudes about e-cigarettes are shown in Table 3. The mean value of the total scale was 2.60 (SD=0.49). Of the five dimensions, the Safety dimension had the highest mean value (Mean=3.34), which was followed by Restriction dimension and Accessibility dimension (Mean=2.66, Mean=2.64, respectively). The lowest mean value occurred for Supervision dimension (Mean=2.00). Similarly, the mean value for Acceptability was only 2.40.

**Table 3:** Attitudes regarding e-cigarettes among university students, Hangzhou, China 2019–2020 (N=563)

*Dimension*	*Total sample Mean (SD)*	*Ever users Mean (SD)*	*Non-users Mean (SD)*	*t*	p*
Accessibility	2.63 (0.75)	2.49 (0.85)	2.64 (0.74)	1.343	0.180
Acceptability	2.40 (0.74)	2.35 (0.95)	2.40 (0.71)	0.365	0.717
Safety	3.34 (0.64)	3.30 (0.90)	3.34 (0.61)	0.268	0.790
Supervision	2.00 (0.79)	2.27 (0.88)	1.98 (0.77)	-2.440	0.015*
Restriction	2.66 (0.83)	2.92 (0.85)	2.64 (0.82)	-2.193	0.029*
Total scale	2.60 (0.49)	2.72 (0.54)	2.59 (0.49)	-1.785	0.075

* p<0.05.

The participants who were ever users of e-cigarettes had more favorable attitudes towards e-cigarettes than the students who were non-users in the Supervision dimension and Restriction dimension (p<0.05).

### E-cigarette use and its correlates

Ever users (who used e-cigarettes at least once in their lifetime) accounted for 8.2% of the students. Of these, ever users who used e-cigarettes at least once in the past year accounted for 7.3%, current non-daily users accounted for 2.0%, current daily users accounted for 1.1%. The other 89.6% of ever users were all former users.

As shown in Table 4, Pearson’s chi-squared tests showed that ever e-cigarette users and non ever users differed by year in school (df=3, p=0.001), major (df=4, p=0.000), urban/rural region (p=0.002), per capita household monthly income (df=2, p=0.000), conventional cigarette use (p=0.000), friends’ ever e-cigarette use (p=0.000), roommates’ ever e-cigarette use (p=0.000), perception of whether e-cigarettes contain nicotine (df=2, p=0.001), perception of whether e-cigarettes are a tobacco product (df=2, p=0.005), and perception of whether e-cigarettes are addictive (df=2, p=0.000).

**Table 4:** Chi-squared test for predicting correlates of ever e-cigarette use, Hangzhou, China 2019–2020 (N=563)

*Variables*	*Ever e-cigarette use*		*χ^2^*	*p*
	*No n (%)*	*Yes n (%)*		
**Gender**			5.593	0.018
Male	200 (88.5)	26 (11.5)		
Female	317 (94.1)	20 (5.9)		
**Ethnic group**			0.123	0.726
Han	494 (91.7)	45 (8.3)		
Ethnic minority	23 (95.8)	1 (4.2)		
**Year in school**			17.067	0.001*
First	169 (97.1)	5 (2.9)		
Second	134 (94.4)	8 (5.6)		
Third	134 (87.6)	19 (12.4)		
Fourth	80 (85.1)	14 (14.9)		
**Major**			35.060	0.000*
Science	116 (96.7)	4 (3.3)		
Engineering	86 (94.5)	5 (5.5)		
Medicine	114 (97.4)	3 (2.6)		
Management science	73 (94.8)	4 (5.2)		
Arts	128 (81.0)	30 (19.0)		
**Father’s educational level**			4.017	0.134
Primary or less	55 (90.2)	6 (9.8)		
Secondary	292 (93.9)	19 (6.1)		
Tertiary	170 (89.0)	21 (11.0)		
**Mother’s educational level**			4.239	0.120
Primary or less	80 (94.1)	5 (5.9)		
Secondary	306 (93.0)	23 (7.0)		
Tertiary	131 (87.9)	18 (12.1)		
**Regions**			9.841	0.002*
Urban	269 (88.5)	35 (11.5)		
Rural	248 (95.8)	11 (4.2)		
**Per capita household monthly income** (RMB)			15.507	0.000*
0–5000	181 (96.8)	6 (3.2)		
5001–10000	220 (92.1)	19 (7.9)		
>10000	116 (84.7)	21 (15.3)		
**Conventional cigarette use**			257.102	0.000*
Yes	25 (39.7)	38 (60.3)		
No	492 (98.4)	8 (1.6)		
**Mothers’ ever e-cigarette use**			2.289	0.130
Yes	4 (66.7)	2 (33.3)		
No	513 (92.1)	44 (7.9)		
**Fathers’ ever e-cigarette use**			1.192	0.275
Yes	38 (86.4)	6 (13.6)		
No	479 (92.3)	40 (7.7)		
**Siblings’ ever e-cigarette use**			2.199	0.138
Yes	24 (82.8)	5 (17.2)		
No	493 (92.3)	41 (7.7)		
**Friends’ ever e-cigarette use**			57.060	0.000*
Yes	44 (67.7)	21 (32.3)		
No	473 (95.0)	25 (5.0)		
**Roommates’ ever e-cigarette use**			27.471	0.000*
Yes	65 (77.4)	19 (22.6)		
No	452 (94.4)	27 (5.6)		
**Do e-cigarettes contain nicotine?**			15.114	0.001*
Yes	209 (87.1)	31 (12.9)		
No	104 (92.0)	9 (8.0)		
Unclear	204 (97.1)	6 (2.9)		
**Are e-cigarettes tobacco product?**			10.756	0.005*
Yes	159 (90.9)	16 (9.1)		
No	199 (88.4)	26 (11.6)		
Unclear	159 (97.5)	4 (2.5)		
**Are e-cigarettes carcinogenic?**			1.240	0.538
Yes	260 (90.6)	27 (9.4)		
No	74 (93.7)	5 (6.3)		
Unclear	183 (92.9)	14 (7.1)		
**Are e-cigarettes addictive?**			35.058	0.000*
Yes	344 (92.5)	28 (7.5)		
No	33 (70.2)	14 (29.8)		
Unclear	140 (97.2)	4 (2.8)		

RMB: 1000 Chinese Renminbi about US$160.

* p<0.01

Multiple logistic regression analyses of ever e-cigarette use indicated that students who come from urban areas were more likely to use e-cigarettes (adjusted odds ratio, AOR=0.40; p=0.016). Besides, the students whose friends and roommates ever vaped were more likely to report e-cigarette use (AOR=8.29; p=0.000 and AOR=4.75; p=0.000, respectively). Finally, ever e-cigarette use was strongly associated with the self-reported attitudes toward e-cigarettes in Supervision dimension. The more favorable the students were towards e-cigarettes, the more likely they were to use them (AOR=1.67; p=0.010). All results are presented in Table 5.

**Table 5:** Bivariate logistic regression analyses of ever e-cigarette use, Hangzhou, China, 2019–2020 (N=563)

*Variables*	*AOR*	*95% CI*	*p*
Attitude to supervision	1.67	1.13–2.46	0.010*
Regions	0.40	0.19–0.84	0.016*
Friends’ ever e-cigarette use	8.29	4.10–16.76	0.000*
Roommates’ ever e-cigarette use	4.75	2.34–9.65	0.000*

AOR: adjusted odds ratio.

* p<0.05.

## DISCUSSION

In our study, the level of knowledge regarding e-cigarettes was not high. Fewer than half of participants (42.6%) were sure that e-cigarettes contain nicotine. In addition, fewer than one-third of students (31.1%) identified e-cigarettes as tobacco products. Additionally, 20.1% participants believed e-cigarettes do not contain nicotine. In fact, 99% of e-cigarettes sold in the United States contained nicotine in 2015^17^, but the manufacturers do not always list accurate nicotine concentrations on e-cigarette labels^18^. With respect to the perception of carcinogenicity, the level of knowledge of the students in our study is nearly 2.5 times higher than those of two universities in Shanghai^12^. However, the percentage of students who were aware of addictiveness in the two different cities was almost the same^12^. Universities play a vital role in prevention of nicotine and tobacco use among students. Therefore, it is important to develop educational and health messages to correct misperceptions about e-cigarettes and raise the level of knowledge about e-cigarettes. It is also recommended that youth receive education in the context of e-cigarette prevention^19^.

It is becoming increasingly prevalent among college students to hold positive attitudes towards e-cigarettes^20^. We found that the participants in our study had the most positive attitudes toward the Safety dimension, indicating that they felt e-cigarettes have less health dangers than conventional cigarettes. In addition, Supervision dimension was least supported by respondents among the 5 domains. In other words, the students are in favor of more supervision on purchasing and vaping among minors. Results also showed that students who used e-cigarettes had significantly more favorable attitudes than non ever e-cigarette users in the dimension of Supervision and Restriction, which were similar to previous findings^1^. The results demonstrated that it is important to regulate e-cigarettes strictly online and offline. Besides, although a ban on e-cigarette online sales has been declared in China, it is advisable to raise the minimum purchasing age for tobacco to 21 years^21^.

In comparison to studies in foreign countries, our investigation reported lower prevalence of ever e-cigarette use and current e-cigarette use. In a study of 1476 New Zealand university students, 40.5% of respondents reported ever e-cigarette use, 6.1% current use, and 1.7% daily use^7^. In Italy, even nursing students had higher rates of vaping than our students^22^. In addition, ever use of e-cigarettes was reported by 13.8% of the 404 undergraduates attending a medical university in Malaysia^23^. However, the prevalence we found of ever e-cigarette use is higher than the estimates reported previously from two universities in Shanghai, China^12^. In 2018 in Shanghai, of 869 university students from two universities, 4.6% of respondents reported ever e-cigarette use, 1.7 % current use, and 0.2% daily use in the previous 30 days^12^. Compared with the survey in Shanghai, our study may have found higher rates because we had 20.8% medical students, while 44.4% were medical students in their study. It was found that non-medical students were more likely to use e-cigarettes than medical students^12^.

Our findings indicate that roommate and friend ever e-cigarette use are associated with greater probability of ever e-cigarette use, which is consistent with another study in China^12^. Moreover, it was found that beliefs about how many peers use e-cigarettes can translate into increased adolescent e-cigarette use^1^ , as social interactions are an important way for sharing information about e-cigarettes and motivating more people to use e-cigarettes^24^. When exposed to e-cigarettes through friends and roommates, university students may be easily attracted by the sweet flavors and novelty^25^. The results of our study also indicate a significant relationship between students coming from urban areas before being enrolled at the university and ever e-cigarette use. Compared with students coming from rural areas, possible influences related to greater likelihood of e-cigarette use for students from urban areas could include advertising, accessibility, or socioeconomic differences^26^. The factor needs to be taken into consideration when conducting education programs. Last but not least, the students who hold more favorable attitudes towards e-cigarettes, especially about supervision, were more likely to use e-cigarettes. As e-cigarettes are becoming a more popular tobacco product among youth, their attitudes towards these products should be taken into consideration in designing tobacco control policies.

### Limitations

There are several limitations in this study. First, participants consisted exclusively of Chinese students from a university, so the generalizability is limited. In the future, surveys with larger sample sizes and conducted in more universities are needed to get a more comprehensive understanding of the latest e-cigarette use status. Second, another main limitation of this study is information bias. For example, some students used e-cigarettes in their lifetime but did not report it in the survey. Compared with face-to-face interviews, online surveys may be more difficult to ensure data quality. Finally, given the research design of our study, we were unable to analyze causal relationships.

## CONCLUSIONS

Our investigation of university students suggests that 8.2% had ever used e-cigarettes and 2.0% were current users, which is lower than studies in foreign countries but higher than other studies in China. Additionally, this study investigated contributing factors of e-cigarettes use, showing that the students who come from urban areas, whose friends were e-cigarette users, whose roommates were e-cigarette users, and who hold more favorable attitudes towards e-cigarettes were more likely to use e-cigarettes.

## Data Availability

The data supporting this research are available from the authors on reasonable request.
