# Supplementary material for: Electronic cigarette knowledge, attitudes and use among students at a university in Hangzhou, China
Source: Tob Induc Dis. 2022 Jan 28;20:09. doi: 10.18332/tid/144230 (PMC8796848; doi:10.18332/tid/144230)
Supplement: Supplementary file 1 [file TID-20-09-s1.pdf]

Appendix 1 Specific items for students' attitudes on e-cigarettes

Dimension	Item
Accessibility	Cigarettes cost less money than e-cigarettes.
	Cigarettes are easier to get than e-cigarettes.
Acceptability	E-cigarettes are more pleasurable than cigarettes.
	Vaping increase young people's appeal.
Safety	The second-hand smoke of e-cigarettes is more harmful than that of cigarettes.
	E-cigarettes are safer than smoking.
	E-cigarettes work as a smoking cessation aid.
	E-cigarettes are trendier and more prevalent than conventional cigarettes.
Supervision	Underage vaping should be banned in public places.
	For minors, e-cigarettes should be regulated as tightly as cigarettes by the government.
	Some e-cigarette products have problems such as oil leakage, poor quality batteries and unsafe ingredients, which are really harmful for youth.
	All types of market entities shall not sell e-cigarettes to minors.
	Any organization and individual should discourage and stop the sale of e-cigarettes to minors.
Restriction	E-cigarettes should be banned online.
	Advertising online for e-cigarettes should be limited or banned.
	There should be a ban on the online sale and marketing of e-cigarettes.
	Enterprises or individuals producing and selling e-cigarettes should be urged to shut down e-cigarette Internet sales websites or clients.
	E-commerce platforms should be urged to remove e-cigarette products and close e-cigarette stores.
	Enterprises or individuals producing and selling e-cigarettes should be urged to withdraw their e-cigarette advertisements posted on the Internet.

© 2022 Fang J. et al.
